# Cancer Therapy-Related Cardiac Dysfunction, Risk Stratification, and Outcomes in ICOP Registry: First Data from the Arabian Middle East Region

**DOI:** 10.5334/gh.1547

**Published:** 2026-03-27

**Authors:** Hasan Ali Farhan, Ali Abdulhameed Ali, Israa Fadhil Yaseen

**Affiliations:** 1 Cardio-Oncology Clinic, Baghdad Medical City, Baghdad, Iraq; 2 University of Baghdad, College of Medicine, Baghdad, Iraq; 3 Scientific Council of Cardiology, Iraqi Board for Medical Specializations, Baghdad, Iraq; 4 Baghdad Heart Center, Baghdad Teaching Hospital, Medical City, Baghdad, Iraq

**Keywords:** Cardio-oncology, CTRCD, CTR-CVT, incidence, Middle East

Despite the evolving cardio-oncology discipline globally, data regarding cardio-oncology in the Arabian Middle East is lacking. The latter issue may be explained by the absence of cardio-oncology clinics in the region over the last few years. In general, data from the Middle East in the field of cardiology were limited, and Iraq was among the first four countries in the region with the lowest publications; only 67 publications until 2015 (1). In 2020, a cardio-oncology clinic was established in Iraq, and two prospective studies were conducted: Iraqi Cardio-Oncology Program (ICOP) and ICOP-Pharm registries (2).

The current study will be the first and largest published data in the field of cardio-oncology from the Arabian Middle East region to identify the incidence of cancer therapy-related cardiovascular toxicities (CTR-CVT), including early cancer therapy-related cardiac dysfunction (CTRCD), with the mean time of left ventricular ejection fraction (LVEF) recovery. CTR-CVT is defined as any cardiovascular adverse events induced by cancer therapy, for example, CTRCD, myocarditis, hypertension, etc. (3) CTRCD is the negative effect of cancer therapy on the heart structure and/or function, leading to either asymptomatic or symptomatic heart failure (3). ICOP registry, a prospective observational study, was conducted at the first cardio-oncology clinic in Iraq between December 2020 and December 2022. All patients attending the clinic were enrolled in the study. The study was approved by the National Research Ethics Committee.

Over 2 years, 638 patients with cancer were included in the study with mean age 55 ± 13 years, most of the patients were women (524 [82%]). Hypertension (244 [38%]) and diabetes mellitus (126 [20%]) were the most common baseline cardiovascular risk factors. The three main causes for referral to the cardio-oncology clinic were patients on cancer therapy (278 [44%]); for baseline cardiovascular risk assessment—either for cardiotoxicity risk stratification using HFA-ICOS risk score or for a request for performing baseline echocardiography—(212 [33%]); and due to cardiac symptoms (87 [14%]).

**Figure 1:**
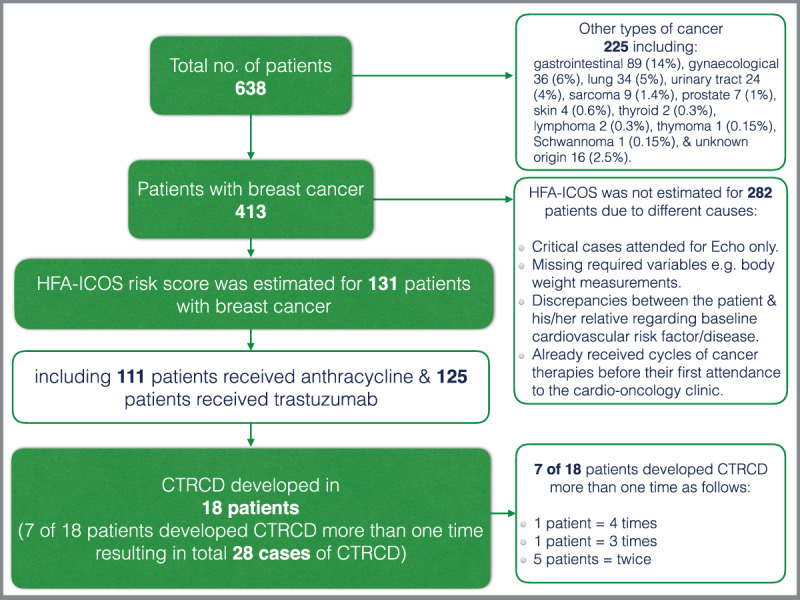
Flow diagram for the studied cohort.

The majority of the patients had breast cancer (413 [65%]), followed by gastrointestinal tract (89 [14%]), and gynecological (36 [6%]) cancers (Figure 1).

CTR-CVT were recorded in 116 (18%) patients, mainly hypertension induced by cancer therapy (40 [10%]) and CTRCD (41 [7%]). Trastuzumab was the most common cause of CTRCD (26 [63%]). Central illustration A.

**Central illustration:**
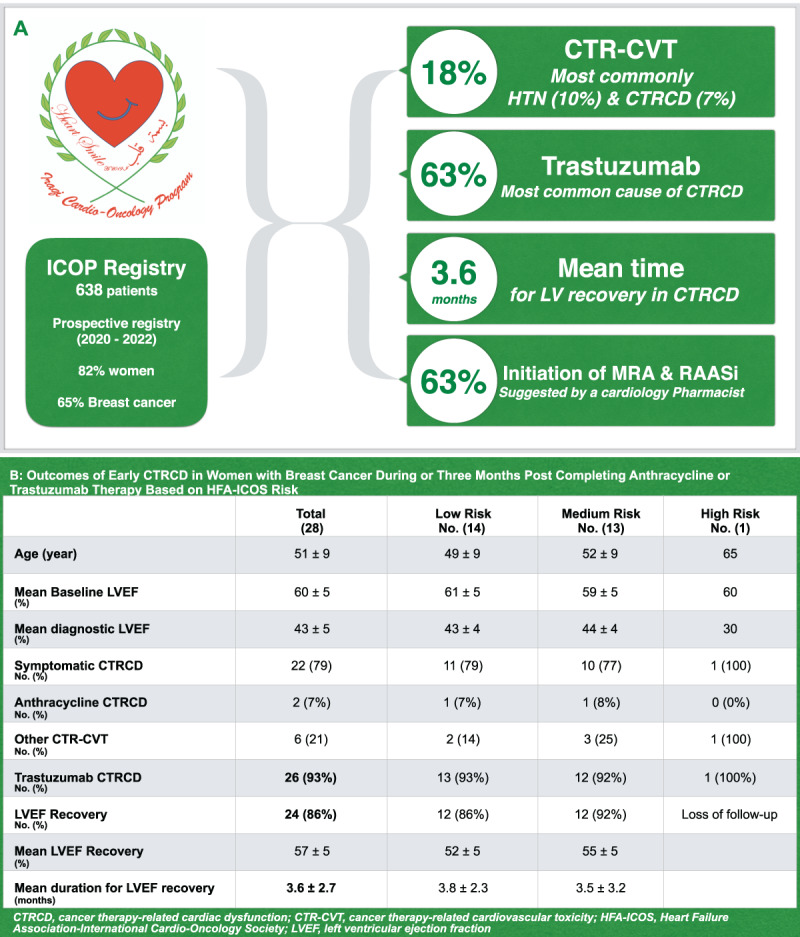
**A.** Outcomes of ICOP registry. **B.** Outcomes of early CTRCD in women with breast cancer based on HFA-ICOS risk stratification.

Among 413 patients with breast cancer, baseline HFA-ICOS risk score was calculated for 131 (32%) patients who received trastuzumab and/or anthracycline. The HFA-ICOS score is an evidence-based tool for baseline cardiovascular risk stratification of oncology patients before starting cancer therapy, known to induce cardiovascular toxicities, leading to improved personalized management of patients with minimization of cardiovascular complications related to cancer therapy. This tool stratifies patients into low, medium, high, and very high risk categories (4). Of note, the HFA-ICOS score was not calculated for all the patients because either they were already receiving some cycles of cancer therapy, emergencies cases, or due to lack of some of the information which are required in the risk estimation. HFA-ICOS scores for the majority of patients who received anthracycline were in low risk (77 [69%]), followed by medium risk (27 [24%]), and high risk (7 [6%]) categories. Of anthracycline risk groups, 1 (1.3%) patient in low risk and 1 (3.7%) patient in medium risk developed CTRCD. While for patients who received trastuzumab, the majority were in medium risk (72 [58%]), followed by low risk (43 [34%]), high risk (9 [7%]), and very high risk (1 [0.8%]). However, most of the patients who developed CTRCD induced by trastuzumab (8 [18.6%]) were in the low risk category compared with patients in the medium risk category (7 [9.7%]). Also, 1 (11.1%) patient in the high-risk group developed CTRCD induced by trastuzumab.

Early CTRCD (defined in the current study as CTRCD development during cancer therapy management or 3 months post completion of cancer therapy cycles) among patients with breast cancer was recorded in 18 patients (28 cases, because some patients developed CTRCD more than one time). LVEF recovery was 24 (86%), including one patient with anthracycline-induced CTRCD, and the mean time for left ventricular function recovery among patients with CTRCD was 3.6 ± 2.7 months. Central illustration B.

Of the total guideline-directed medical therapy (GDMT) prescribed for the management of CTRCD, the cardiology pharmacist suggested the initiation of 15 of 24 (63%) renin–angiotensin–aldosterone inhibitors (RAASi), including angiotensin converting enzyme inhibitors (ACEi), angiotensin receptor blockers (ARB), and angiotensin receptor-sacubitril inhibitors (ARNI), 10 of 16 (63%) mineralocorticoid receptor antagonists, 13 of 23 (57%) β blockers, and 1 of 3 (33%) sodium-glucose transporter-2 inhibitors (SGLT2i).

Regarding CTR-CVT, hypertension and CTRCD were the top two cardiotoxicities developed over 2 years among patients with cancer. For patients with breast cancer who developed CTRCD, the majority of the patients were in the low and medium risk category according to the HFA-ICOS risk score. However, most of the patients with trastuzumab-induced CTRCD were in the low risk rather than medium risk category. The latter finding indicates the need for validation of the HFA-ICOS risk score. Although CARDIOTOX registry showed a positive correlation between HFA-ICOS risk score for developing cardiotoxicity and mortality, it was only conducted for anthracycline use rather than trastuzumab (5). Of note, there was no significant difference between patients in the low and medium risk categories.

Despite previous evidence about the irreversible cardiotoxicity induced by anthracycline, emerging data support the possible reversibility, particularly with the availability of new GDMT such as ARNI and SGLT2i in addition to long-term follow-up studies (6,7,8). On the other hand, although it is known that CTRCD induced by trastuzumab is reversible, it was reported to be associated with irreversible cardiotoxicity (9,10,11). This may explain the reason behind failure of the LVEF recovery among some of our patients who developed CTRCD induced by trastuzumab. Time for LVEF recovery in that study was shown to be at 6-month follow-up, longer than documented in our study, this may be related to the use of ACEI/ARB and β blocker only, unlike in the current study, in which MRA and SGLT2i, were also used (11). In the current study, enrolment of cardiology clinical pharmacists in the cardio-oncology heart team led to improvement in the initiation of GDMT. The significant role of pharmacists in optimization of GDMT, including the addition of MRA, was shown previously in a prospective study (12).

In summary, the current study is the first and largest evidence-based and real-world practice from the Arabic Middle East region in the field of cardio-oncology. It showed that hypertension and CTRCD were the most common CTR-CVTs incident among patients with cancer. Trastuzumab was the leading cause for CTRCD with patients at the low risk category representing the majority of the patients rather than at the medium risk category. Mean time of LVEF recovery after CTRCD was <4 months. The role of cardiology pharmacist was important to improve the management of CTRCD, particularly in optimizing the use of MRA and RAASi.
